# The HYSTER study: the effect of intracervically administered terlipressin versus placebo on the number of gaseous emboli and fluid intravasation during hysteroscopic surgery: study protocol for a randomized controlled clinical trial

**DOI:** 10.1186/s13063-018-2442-9

**Published:** 2018-02-14

**Authors:** Lucilla E. Overdijk, Bart M. P. Rademaker, Paul J. M. van Kesteren, Peter de Haan, Robert K. Riezebos, Oscar C. H. Haude

**Affiliations:** 1 OLVG Hospital, Oosterparkstraat 9, 1091 AC Amsterdam, Netherlands; 2 University of Amsterdam/AMC Hospital, Meibergdreef 9, 1105 AZ Amsterdam, Netherlands

**Keywords:** Hysteroscopic surgery, Transcervical resection of myomas, Transcervical resection of endometrium, Intravasation, Terlipressin, Emboli, Echocardiography

## Abstract

**Background:**

Transcervical resection of myoma or endometrium is a safe, hysteroscopic, minimally invasive procedure. However, intravasation of distension fluid is a common phenomenon during these procedures. In a previous study we observed venous gas emboli in almost every patient. The severity of hysteroscopic-derived embolization has been shown to be correlated to the amount of intravasation. In addition, paradoxical gas embolism, which is potentially dangerous, was observed in several patients.

Studies have shown a reduction of intravasation by using intracervically administered vasopressin during hysteroscopy. We think that its analog, terlipressin, should have the same effect. In our previous research we observed more gaseous emboli as intravasation increased. Whether or not the insertion of intracervically administered terlipressin leads to a lower incidence and severity of gas embolism is unknown. We hypothesize that intracervically administered terlipressin leads to a reduction of intravasation with a lower incidence and severity of gas embolism. Terlipressin may be of benefit during hysteroscopic surgery.

**Methods/design:**

Forty-eight patients (ASA 1 or 2) scheduled for transcervical resection of large, types 1–2 myoma or extensive endometrium resection will be included. In a double-blind fashion patients will be randomized 1:1 according to surgical treatment using either intracervically administered terlipressin or placebo. Transesophageal echocardiography will be used to observe and record embolic events. A pre- and post-procedure venous blood sample will be taken to calculate intravasation based on hemodilution. Our primary endpoint will be how terlipressin influences the severity of embolic events. Secondary endpoints include the effect of terlipressin on the amount of intravasation and on hemodynamic parameters.

**Discussion:**

If terlipressin does indeed reduce the number of gaseous emboli and intravasation occurring during hysteroscopic surgery, it would be a simple method to minimize potential adverse events. It also allows for prolonged operating time before the threshold of intravasation is reached, thereby reducing the need for a second operation.

**Trial registration:**

Nederlands Trial Register (Dutch Trial Register), ID: NTR5577. Registered retrospectively on 18 December 2015.

**Electronic supplementary material:**

The online version of this article (10.1186/s13063-018-2442-9) contains supplementary material, which is available to authorized users.

## Background

Surgical hysteroscopy is an established, minimally invasive procedure. Its advantages consist of a short operating time, rapid post-operative recovery and low morbidity. Although risks and complications during hysteroscopy are rare [1–3] serious complications include fluid overload by intravasation of large amounts of distension fluid and the occurrence of air or gaseous emboli which can be potentially life threatening [3–12]. During hysteroscopic surgery large uterine veins are exposed and may, therefore, be an entry point for gas, air and distension fluid. Most of the symptoms that occur during such an event have either been attributed to the direct effects of venous emboli, such as air lock in the right ventricle (RV), or to the resulting cardiovascular collapse [4]. However, we also observed paradoxical embolism with the passage of venous air or gas into the arterial circulation [13]. This may account for some of the described cardiovascular and neurological symptoms associated with large venous emboli. Indeed, one case of temporary blindness has been associated with paradoxical embolism [10].

In a recent study we observed venous embolism in almost all patients (98%) using either monopolar or bipolar diathermia [14]. In addition, in two patients paradoxical embolism was seen, which raised concern because of its potentially deleterious effects on vital organs. A positive correlation was found between the amount of intravasation and the grade of venous gas embolism.

Guidelines indicate that transcervical surgery should be ceased when intravasation exceeds 2000 ml [15]. For this reason some patients will have to undergo a second operation for removal of the remaining uterine tissue.

Vasopressin and terlipressin (triglycyl-lysine vasopressin) are commonly used drugs with different indications. Vasopressin is a naturally occurring hormone and is secreted by the posterior lobe of the pituitary gland. In some European countries vasopressin is not available and, thus, terlipressin, a synthetic long-acting analog of vasopressin, is used. Owing to its pronounced vasoconstrictive effect within the splanchnic circulation, terlipressin is widely used to treat patients suffering from variceal bleeding during the treatment of hepatorenal syndrome and catecholamine-unresponsive septic shock.

In gynecologic surgery, vasopressin has been used since the 1950s. The utilization of intracervically administered vasopressin has been shown to limit the amount of bleeding and intravasation by its primary effect of vasoconstriction and, therefore, may be of use during hysteroscopic surgery [9, 12, 16–20]. In the Netherlands it is common gynecological practice to use terlipressin intracervically to control bleeding during conization. Because previous studies have shown a reduction of intravasation by vasopressin during hysteroscopy we think that its analog, terlipressin, should have the same effect. Terlipressin is an authorized and licensed product used by many clinicians and its efficacy is supported by sufficient published evidence. Whether intracervically administered terlipressin use leads to a decrease in the incidence and severity of gas embolism and the amount of intravasation is unknown and will be the subject of investigation during this phase IV study. In addition, we will study the effects of terlipressin and gaseous emboli on hemodynamic parameters.

## Methods/design

### Objective

The primary objective of the study is to determine whether the intracervical utilization of terlipressin reduces the incidence and severity of gas embolism as detected by transesophageal echocardiography (TEE).

The secondary objective is to study the effects of the intracervical utilization of terlipressin on the amount of intravasation, global hemodynamics, myocardial ventricular systolic strain and myocardial diastolic function using TEE.

### Sample size calculation

In a recent study we observed venous embolism in 98% of the patients undergoing hysteroscopic surgery using TEE. In addition, a positive correlation has been found between the amount of intravasation and the number of venous emboli. We consider it likely that venous emboli will be observed in all patients undergoing transcervical resection of myomas or endometrium (TCR-M or TCR-E, respectively) using bipolar diathermia. Previous studies have shown a 45–65% reduction in the amount of intravasation using intracervically administered vasopressin [17]. Assuming a similar rate of reduction in emboli, we have calculated our sample size keeping in mind that there will be 45% less intravasation while using terlipressin. Power analysis reveals that 24 patients need to be included in both groups in order to achieve an alpha value of 0.05 assuming that terlipressin does not result in a 45 to 65% reduction of venous emboli.

### Study population

Forty-eight patients (ASA classification 1 or 2) scheduled for transcervical resection of large, types 1–2 myomas or extensive TCR-E will be included. All patients are recruited and treated in the OLVG Hospital in Amsterdam.

Exclusion criteria include patients not expected to benefit from diminished intravasation due to terlipressin utilization. These include patients undergoing transcervical operations for small myomas and minor TCR-E procedures. Procedures that are expected to be short lasting (less than 30 min) are excluded.

Patients with a known contraindication for TEE, such as severe esophageal or gastric disease, hepatic cirrhosis or esophageal varices, will be excluded from the study. In addition, patients aged < 18 years or > 70 years, patients with a history of pulmonary embolism or cardiac disease and patients with a language barrier will be excluded.

Eligible patients are informed of the study by gynecologists and anesthesiologists in the outpatient clinic. All patients receive oral and written information about the study. Final inclusion is always done by one of the researching anesthesiologists. If informed consent is obtained randomization will be done on the day of surgery. From that moment on collection of data starts.

### Study medication

The patients in our study will be treated with either intracervically administered terlipressin (Glypressine®, Ferring Pharmaceutical) or placebo. Terlipressin treatment will consist of 20 ml diluted terlipressin at a concentration of 0.0425 mg/ml (one vial Glypressine® 8.5 ml containing 0.1 mg/ml terlipressin diluted with 11.5 ml 0.9% saline (NaCl)). The placebo treatment consists of 20 ml 0.9% saline. The maximum dose of terlipressin administered intracervically will be 0.85 mg. The investigational product will be used in the same way as is commonly used in conization: at the start of the procedure patients will receive a maximum of 20 ml investigational product intracervically injected clockwise in the cervix at four points.

### Randomization, blinding and treatment allocation

Randomization for intracervically administered terlipressin or placebo will be performed by our hospital pharmacologist, using blocked randomization in blocks of eight patients with a standard randomization scheme, on a 1:1 ratio. The blinded vials (containing 20 ml diluted terlipressin or 20 ml placebo) will be delivered at the day of surgery to the operating team. Echocardiographic images will be evaluated off line by a trained observer, who is also blinded for the treatment group.

### Main study endpoints

The main study parameter is the appearance of any embolic event, either venous or paradoxical in origin. To quantify the degree of embolism by TEE a five-grade scale is used:

- Grade 0: the absence of emboli passing through the heart
- Grade I: the presence of a limited number (<10 per field of view) of small particles in the right atrium (RA), right ventricle (RV) and right ventricular outflow tract (RVOT)
- Grade II: a moderate number of small particles (10–20 per field of view)
- Grade III: many small particles (>20 per field of view)
- Grade IV: many small and large particles (>20 per field of view) completely filling the diameter of the RA, RV and RVOT

### Secondary study endpoints

The secondary endpoints collected during the study are:

1. Hemodynamic parameters: blood pressure, cardiac output and systemic vascular resistance
2. Systolic and diastolic cardiac function
3. Signs of cardiac ischemia
4. Amount of intravasation

These endpoints are measured as follows:

1. Hemodynamic parameters will be measured using standard monitoring and with a non-invasive, beat-to-beat hemodynamic monitor (Nexfin HD, BMEYE B.V, Amsterdam, Netherlands)
2. Measurements of systolic and diastolic cardiac function will be assessed by TEE before and just after surgery:
  - TEE-derived two-dimensional (2D) strain analysis using speckle-tracking (GE healthcare, EchoPAC™) of longitudinal left and right ventricular systolic strain will be made in each of the three mid-esophageal, longitudinal planes: mid-esophageal, four-chamber view (ME 4ch), mid-esophageal, two-chamber view (ME 2ch), and mid-esophageal, long-axis view (ME LAX)
  - Using pulsed-wave Doppler the transmitral flow pattern (E/A ratio) will be recorded for the assessment of left ventricular diastolic function
  - The diastolic function will be assessed by the ratio of E/e’. The e’ is measured by tissue Doppler imaging in the basal lateral walls of the mid-esophageal, four-chamber view. The mean e’ will be used for the analysis
3. To exclude myocardial ischemia or damage a continuous, five-lead electrocardiogram (ECG) with ST segment analysis, a post-operative, 12-lead ECG and a high-sensitivity troponin analysis are used
4. Intravasation will be measured by the surgical team by subtracting the amount of fluid introduced via the resectoscope and the amount of fluid collected by fluid suction

Furthermore, intravasation will be calculated by the difference between pre- and post-procedure hematocrit (Ht) and hemoglobin (Hb) concentration according to the formula reported by Drobin et al. [21] and Johansson et al. [22]:

- (Baseline Hb/Hb after procedure − 1) x plasma volume/1 − baseline Ht
- (ECFV × postop[Cl] − ECFV × preop[Cl])/(154 − postop[Cl]),

where ECFV = extracellular fluid volume, postop = postoperative, preop = preoperative, Cl = plasma chloride concentration

### Study procedures

1. A 5-mHz multiplane TEE probe (Vivid-i GE Vingmed Ultrasound AS, Horten, Norway) will be inserted under general anesthesia after tracheal intubation into the esophagus to obtain a four-chamber view. TEE images will be recorded at set times and all recordings will consist of three cardiac cycles. For baseline measurements the first TEE recording will be made after the insertion of the probe, before installation of terlipressin/placebo and before the start of the surgery. To obtain global strain as an overall measure of ventricular systolic function the three mid-esophageal, longitudinal planes will be viewed: ME 4ch, ME 2Ch, ME LAX
2. Before installation of terlipressin/placebo a venous blood sample is taken
3. The blinded study medication is injected intracervically
4. Two minutes after intracervical injection of the study medication Nexfin-derived hemodynamic variables will be re-recorded
5. During transcervical surgery TEE images will be made throughout the whole procedure. Images are made when emboli appear on a regular basis with a minimum of 10 loops during the whole procedure. The total duration of embolic events will also be recorded
6. The amount of intravasation will be recorded at the end of hysteroscopic surgery
7. After surgery final TEE recordings will be made. To obtain global strain measures after surgery as an overall measure of ventricular systolic function, the three mid-esophageal, longitudinal planes will be viewed again
8. After surgery final hemodynamic recordings will be made using the Nexfin HD
9. After the procedure and final measurements the second venous blood sample is taken
10. In the recovery room a 12-lead ECG is performed
11. Three hours after surgery a third blood sample will be taken for high-sensitivity troponin analysis

**Fig. 1:**
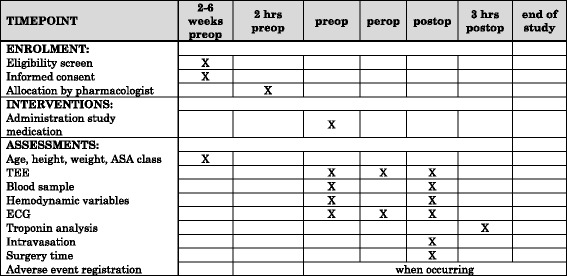
SPIRIT figure HYSTER: Schedule of enrolment, interventions and assessments for HYSTER study. *Preop* preoperative, *perop* peroperative, *postop* postoperative, *TEE* TransEsophageal Echocardiography

The SPIRIT figure HYSTER (Additional file 1) shows in detail the schedule of enrollment, interventions and assessments. For flow diagram HYSTER study see (Fig. 1).

### Statistical analysis

- *Descriptive statistics*: data are presented in tables and graphs as totals, means, medians, percentiles and percentages when indicated
- *Multivariate analysis*: continuous variables (demographic data, intravasation, etc.) will be analyzed using the unpaired *T* test or the Mann-Whitney *U* test. Non-parametric data will be analyzed using the chi-square test. Global hemodynamic data (heart rate, cardiac output, etc.) will be analyzed using multivariate analysis of variance (MANOVA) for repeated measures. Post hoc analysis will be done using Dunnett’s post hoc test. Non-parametric data, i.e., grading of the embolic events, will be analyzed using the Wilcoxon signed-rank test. A *p* value ≤ 0.05 will be considered statistically significant. All statistical analysis will be performed using SPPS (version 22.0.0.1)
- *Univariate analysis*: not applicable

### Data management

Paper, patient records are stored in a designated locker, only accessible to authorized researchers. Electronic patient data are either stored in the hospital electronic health record system or on a password-secured local Sharepoint (Microsoft) server, also only accessible to authorized researchers. Individual files containing patient data are password protected separately. Researchers with access to patient data are BROK-certified. BROK (Basic course on Regulations and Organization for clinical investigators) is a legally required certificate for academic researchers issued by the Dutch Federation for University Hospitals.

Every included patient is allocated a personal study number. If an unexpected finding is discovered requiring further examination (for example, during offline analysis of echocardiography) blinded data can be traced back to individual patients.

### Harms

Adverse events, serious adverse events and suspected unexpected serious adverse reactions (AEs, SAEs and SUSARs, respectively) related to the hysteroscopic procedure will be treated according to generally accepted guidelines. Firstly, if needed, the hysteroscopic procedure is stopped. AEs and SAEs related to the installation of the TEE probe (esophageal lacerations, bleeding, etc.) will be treated following consultation with a gastroenterologist and a surgeon. In case of serious cardiac adverse events patients will be treated following consultation with a cardiologist. Owing to its pronounced vasoconstrictive effect within the circulation, the use of terlipressin may result in hypertension with or without reflex bradycardia. This will be treated, if necessary, with the appropriate drugs commonly used in general anesthesic practice Preventive measures in this respect are a proper injection technique (aspiration before injection in order to prevent intravascular injection) and extensive monitoring of heart rate and blood pressure. A 24-h Emergency Code Break and Drug Information can be provided by the hospital pharmacist.

SAEs and SUSARs are always reported to the involved Research Ethics Committee. All AEs, SAEs and SUSARs will be followed up until they have abated, or until a stable situation has been reached. Depending on the event, follow-up may require additional tests, medical procedures, and/or referral to the general physician or a medical specialist. Interim analysis will be done after 25 patients have been included.

As legally required a liability insurance covers potential physical harm inflicted to patients through study procedures.

## Discussion

This protocol describes a double-blind randomized trial to compare the effect of intracervically administered terlipressin with placebo in regard to the degree of gaseous embolism and intravasation during hysteroscopic surgery. If terlipressin reduces gaseous embolism and intravasation it would be a potentially simple intervention to make hysteroscopic surgery an even safer procedure. As previously stated, minimizing gaseous emboli and intravasation might reduce the need for a second operation.

This study does not only investigate the possible benefits of intracervically administered terlipressin, we also study the effects of terlipressin itself on hemodynamic parameters and specific cardiac parameters using TEE.

The results of pre- and post-procedure venous blood gas samples enable the future analysis of acid-base and electrolyte disturbances relating to intravasation during hysteroscopic surgery.

## Trial status

At the time of writing this manuscript, 45 patients have been included and treated according to the protocol. The first patient was included April 2014; recruitment is expected to be completed around December 2017.

## Additional file

Additional file 1: SPIRIT Checklist HYSTER. SPIRIT Checklist HYSTER shows the recommended items to address in a clinical trial protocol with corresponding page numbers. (DOC 122 kb)

## Abbreviations

AE: Adverse event
A**S**A: American Society of Anesthesiologists
Hb: Hemoglobin
Ht: Hematocrit
ME 2ch: Mid-esophageal, two-chamber view
ME 4ch: Mid-esophageal, four-chamber view
ME LAX: Mid-esophageal, long-axis view
NaCl: Sodium chloride solution or saline
RA: Right atrium
RV: Right ventricle
RVOT: Right ventricular outflow tract
SAE: Serious adverse event
SUSAR: Suspected unexpected serious adverse reaction
TCR-E: Transcervical resection of endometrium
TCR-M: Transcervical resection of myomas
TEE: Transesophageal echocardiography
